# Detection of Shamonda Virus in Two Horses with Neurological Signs in Germany

**DOI:** 10.3390/v18101125

**Published:** 2026-10-10

**Authors:** Marielle Elen Volkwein, Judith Köster, Ernst Großmann, Nathalie Hafner, Thomas Miller, Andreas Salditt, Petra Benz, Martin Beer, Angele Breithaupt, Kerstin Wernike

**Affiliations:** 1 Aulendorf State Veterinary Diagnostic Centre, Löwenbreitestraße. 18/20, 88326 Aulendorf, Germany; 2 Animal Disease Fund Baden-Württemberg, Horse Health Service Aulendorf, Talstraße 17, 88326 Aulendorf, Germany; 3 Institute of Diagnostic Virology, Friedrich-Loeffler-Institut, Südufer 10, Insel Riems, 17493 Greifswald, Germany; 4 Department of Experimental Animal Facilities and Biorisk Management, Friedrich-Loeffler-Institut, Südufer 10, Insel Riems, 17493 Greifswald, Germany

**Keywords:** *Orthobunyavirus*, Shamonda virus, horse

## Abstract

Shamonda virus (SHAV), which has recently emerged in Central Europe and has caused disease in numerous cattle herds, was detected in brain tissue from two horses that showed clinical signs, including neurological disorder, and underwent pathological examination. The horses reportedly exhibited seizure-like episodes (Horse A) as well as fever and ataxia (Horse B). In both horses, severe lymphohistiocytic meningoencephalitis with hemorrhages was observed in various parts of the central nervous system (CNS), while outside the CNS no abnormalities were found. The virus genome could be detected by RT-qPCR in all examined parts of the CNS of both horses. In a postmortem cerebrospinal fluid sample of one horse, the virus could only be detected with a very low genome load; however, postmortem contamination cannot be completely ruled out. Consistent with the histological findings, no SHAV genome was detected in either the blood or organs outside the CNS. Postmortem, CNS material—especially brainstem tissue—is suitable for detection of histological lesions and virus genomes via RT-qPCR, while intravital diagnostics of a SHAV infection in horses exhibiting neurological signs is at the moment only possible to a very limited extent. The epidemiological role of horses in the SHAV outbreak is currently unclear.

## 1. Introduction

Since mid-June 2026, Shamonda virus (SHAV), an *Orthobunyavirus* belonging to the Simbu serogroup and the species *Orthobunyavirus schmallenbergense*, has emerged in Europe and has been detected in numerous herds of sick cattle [1,2]. Two distinct events of SHAV incursion were identified: SHAV clade Europe 1 (SHAV-EU1) circulating primarily in France, Switzerland and southern Germany and SHAV clade Europe 2 (SHAV-EU2) circulating mainly in the northwest of Germany [3]. As for most other viruses of the Simbu serogroup, SHAV is transmitted by Culicoides biting midges [1,4]. Since June 2026, mild clinical signs such as fever and diarrhea have been reported in cattle, as well as a marked decrease in milk yield in dairy cows [2]. In addition, SHAV has been detected in sheep, goats, and New World camelids, although the clinical outcome in these species remains unknown [5,6]. Soon after the virus was detected in European cattle, the Universities of Bern and Zurich reported six horses with neurological signs in which SHAV was detected in brain tissues [7]. Similarly, SHAV was detected in a horse with neurological abnormalities in the German federal state of Rhineland-Palatinate [5]. Before these, no cases of SHAV in horses had been reported. However, Shuni virus (SHUV), also a member of the Simbu serogroup, has been associated with neurological disease in both horses and cattle [8,9]. Currently, the data available on postmortem and intravital diagnosis of SHAV in horses is very limited. Scientific reports about the pathohistological changes in horses are missing, as are images of lesions or data on the tissues in which SHAV could be detected. SHAV-specific commercial antibody tests are also not yet available.

Here we report two cases of horses with severe neurological signs in which SHAV was detected, providing further insights into the susceptibility and clinical presentation of SHAV in horses.

## 2. Materials and Methods

### 2.1. Case Description

In early September 2026, two horses from different stables, located approximately 37 km apart (measured by straight-line distance) in southern Germany, were submitted for pathological examination to determine the cause of disease. Horse A was a 28-year-old Shetland pony mare that reportedly broke out of the stable the night before, fell into a creek, and was rescued from the creek with visible head injuries. That same evening, a seizure-like episode was observed in the stable, and the pony was euthanized.

Horse B was a 21-year-old mixed-breed pony mare who presented with apathy, teeth chattering, and fever (39 °C). She was treated with meloxicam, cortisone, and an antibiotic. Over the next two days, her general condition continued to deteriorate. The pony exhibited balance problems and head pressing and was euthanized three days after the onset of neurological signs. Both horses were kept in open stable housing, and their owners did not observe any neurological signs prior to the onset of the illness described above. No other horses from the two farms had shown neurological signs so far (four weeks after the euthanasia of Horse A and Horse B).

### 2.2. Laboratory Investigations

Pathological–anatomical and histopathological examinations were performed on both horses. For the histopathological examination, samples of all organs measuring approximately 1 × 1 × 1 cm, sections of the spinal cord in their entirety, one half of the brain in its entirety, and pieces measuring approximately 2 × 2 × 2 cm from the contralateral side of the brain were fixed in 10% buffered formalin for at least two days and subsequently embedded in paraffin, processed routinely for histologic examination, and stained with hematoxylin and eosin (H.E.). In addition, organs were taken for further examination. For each horse, cerebrum, brainstem, and cerebellum were removed and pooled. For Horse B, atlanto-occipital cerebrospinal fluid was collected postmortem and prior to necropsy. In addition, the cerebrum, cerebellum, brainstem, spinal cord, heart, lungs, liver, spleen, and kidneys were individually collected, as well as whole blood in anticoagulant (EDTA) and serum. Following nucleic acid extraction using the IndiMag Pathogen Kit (Indical Bioscience, Leipzig, Germany) according to the manufacturer’s instructions, the samples were tested using a modified version of the Sathu-N3-RT-qPCR assay described by Tauscher et al. [10], which enables the detection of SHAV, Sathuperi virus, and Schmallenberg virus (SBV). To exclude SBV, the only Simbu serogroup virus circulating in Central Europe prior to the emergence of SHAV, the samples were tested with the virotype SBV 2.0 RT-PCR Kit (INDICAL BIOSCIENCE GmbH, Leipzig, Germany) according to the manufacturer’s instructions. The pooled brain samples were additionally tested by a commercial RT-qPCR kit offered for the detection of the SHAV clade circulating in southern Germany, Switzerland, and France (SHAV-EU1) (ID Gene™ SHLV Duplex, Innovative Diagnostics, Grabels, France). Other potential pathogens that can cause neurological signs, like equine herpesvirus 1, equine herpesvirus 4, Borna disease virus 1, West Nile virus, and tick-borne encephalitis virus, were tested as differential diagnoses by PCR as previously described [11,12,13,14].

Moreover, the cerebrum of Horse B was subjected to bacteriological examination; for this purpose, the cerebrum was incised using sterile instruments, and a sample was taken from deep within. This sample was spread onto blood agar and Gassner agar using a three-loop smear technique, incubated aerobically at 38 °C for 3 days, and then assessed macroscopically.

Furthermore, the serum of Horse B was tested for the presence of antibodies using the N-protein-based ID Screen Schmallenberg virus Competition Multi-species ELISA Kit (Innovative Diagnostics, Grabels, France), which enables the detection of antibodies to several members of the Simbu serogroup. Photometric measurements were performed using a Tecan Sunrise microplate reader, and samples with an S/N% value of ≤40% were considered positive, according to the manufacturer’s instructions.

To obtain additional data, the available results from samples taken since early August from cattle on farms in the vicinity of the affected horses, which exhibited clinical signs typical of SHAV, such as fever, diarrhea, or decreased milk production, were analyzed in order to determine a connection between SHAV in cattle and the two cases in horses. For this purpose, the municipalities where the horse farms were located, as well as the neighboring municipalities, were analyzed, as they represent clearly defined geographical and administrative units. SHAV RT-qPCR results used for the analysis were obtained using the same methodology as described above for the horses.

## 3. Results

### 3.1. Autopsy

Horse A was obese (body condition score (BCS) = 7), and Horse B was in good body condition (BCS 5) [15]. Both horses exhibited severe, acute, multifocal skin erosion on the skull with subcutaneous hematomas. The skull vault was intact and unremarkable in both animals. In Horse A, the brain was macroscopically unremarkable, and the spinal cord was not examined. In Horse B, acute, multifocal, petechial hemorrhages were found in the area of the brainstem. Other parts of the brain were macroscopically unremarkable. Acute, extensive unilateral hemorrhages primarily in the substantia grisea were found in the initial segment of the thoracic spinal cord (Figure 1a,b).

In both horses, the stomach was empty, and the intestinal content was scarce. No macroscopically abnormal changes were found in the other organs.

### 3.2. Histopathological Examination

Histopathological examination of brainstem tissue of both horses revealed multifocal, severe, subacute lymphohistiocytic perivasculitis with expansion of the Virchow-Robin spaces by many lymphocytes and few (Horse B) to many (Horse A) macrophages that multifocally extend into the surrounding parenchyma (Figure 1c,d). Oligofocal, mild to moderate, subacute hemorrhages were found adjacent to the blood vessels with partial resorption by macrophages (Horse A). Multifocally, some lymphocytic infiltrates were detectable in the vessel wall, accompanied by edema of the surrounding tissue as well as oligofocal, perivascular accumulation of homogeneous eosinophilic proteinaceous material, consistent with plasma protein extravasation (Figure 1b). Multifocally, there were glial nodules with focal aggregation of glial cells, with overall increased numbers of glial cells and neuronophagia (Figure 1c,d). In horse A, a moderate number of plasma cells were also present in the inflammatory infiltrates. Similar but less severe lesions were observed in the cerebrum (Figure 1e).

In the cerebellum of both horses, multifocal, mild- to moderate lymphohistiocytic perivasculitis, partially extending into the parenchyma, was observed. In addition, multifocal (Horse B) to diffuse (Horse A), mild (Horse B) to moderate (Horse A) subacute lymphohistiocytic meningitis was detected in the cerebellum (Figure 1e). In Horse A, multifocal, mild, subacute lymphohistiocytic meningitis was also observed in the cerebral cortex. The spinal cord was examined only in Horse B, and changes could be detected in all examined segments (cervical, thoracic, and lumbar), consistent with a multifocal, mild acute to subacute lymphohistiocytic vascular and perivascular infiltration, with massive extravasation of erythrocytes, particularly in the areas where hemorrhages were observed macroscopically (Figure 1b). In these areas, vessels with mild acute lymphohistiocytic infiltrates but no hemorrhages were also identified, as well as hemorrhages without adjacent vessels affected by lymphohistiocytic infiltrates. In addition, oligofocal glial nodules in the cervical segment of the spinal cord were detected. Similar, albeit less pronounced, inflammatory lesions were also found in the lumbar segment.

No relevant histopathological changes were observed in the other organs examined (heart, lungs, liver, kidneys, and spleen), except for diffuse, acute, moderate hyperemia and multifocal, acute, mild alveolar edema in the lungs.

### 3.3. Further Diagnostic Testing

For both horses, the pooled brain material tested positive by the Sathu-N3-PCR RT-qPCR but negative by the virotype SBV 2.0 RT-PCR Kit. In Horse B, the cerebrum, cerebellum, brainstem, spinal cord, and cerebrospinal fluid also tested positive by Sathu-N3-PCR RT-qPCR; the results are shown in Table 1.

In addition, the brain pool samples tested positive with the SHAV-specific ID Gene™ SHLV Duplex RT-qPCR with cq values of 23.02 (Horse A) and 24.70 (Horse B), respectively.

The serum of Horse B was additionally tested for antibodies by ELISA (ID Screen^®^ Schmallenberg Virus Competition, Innovative Diagnostics, Grabels, France) and yielded a positive result with an S/N% value of 7.8%.

### 3.4. Differential Diagnostics

As part of differential diagnostics, PCR testing ruled out equine herpesvirus 1, equine herpesvirus 4, Borna disease virus 1, West Nile virus, and tick-borne encephalitis virus as potential causes of disease in both horses. In addition, bacteriological examination of the brain of horse B revealed no bacterial growth.

### 3.5. Additional Data Collected from the Study Region

Since the first detection of SHAV in Germany, numerous samples, primarily from cattle, have been submitted for testing from the region where both horses were kept. Based on these samples, we were able to confirm the occurrence of SHAV-positive cases in cattle in the vicinity of the two equine cases described here. These data were used to better estimate the timeline and the possible duration from infection to onset of the clinical signs (see Supplementary Material). Samples from 141 cattle at 46 farms in the vicinity of Horse A, as well as samples from 35 cattle at 9 farms in the vicinity of Horse B, tested positive for SHAV.

In southern Germany, the first cattle blood samples in which SHAV was detected were collected in late July [1]. However, given that clinical signs had already been reported before the first confirmed detection of the virus, circulation in the region might have begun earlier. In the vicinity of the stable of Horse A, SHAV infections in cattle were detected between 29 and 8 days before the horse was euthanized. In the vicinity of the stable where Horse B was kept, SHAV infections in cattle were detected between 30 and 21 days before the horse was euthanized (Supplementary Material).

## 4. Discussion

Based on the exclusion of other viral and bacterial pathogens known to cause CNS disorders in horses and the detection of SHAV-EU1 in the affected tissue with high viral genome loads, SHAV is the most likely cause of the disease in both horses. However, further studies are needed to strengthen the association between the CNS lesions and virus detection, for example, using RNA in situ hybridization.

In Horse B, extended testing of various tissue samples was performed and showed that SHAV was limited to the central nervous system. The cerebrospinal fluid tested only weakly positive, which is consistent with reports from Switzerland, where the pathogen could not be detected in the cerebrospinal fluid [7]. It should also be noted that the cerebrospinal fluid was collected postmortem; therefore, contamination resulting from the decomposition of CNS tissue cannot be ruled out. As reported from the cases in Switzerland, SHAV could not be detected in the blood of both horses at the time point of sample collection [7]. This is consistent with other equine neurotropic arboviruses, such as WNV or TBEV (tick-borne encephalitis virus), in which severe neurological disorder typically develops after the viremic phase has subsided [16,17,18]. In addition, the closely related SBV induces only a very short viremia of a few days in its target species [19]. A similarly transient viremic phase may also occur during SHAV infection in horses and could provide a plausible explanation for the diagnostic findings observed in animals with encephalitis.

Furthermore, antibodies were detected in Horse B using the commercially available ID Screen Schmallenberg virus Competition Multi-species ELISA Kit, which enables the detection of antibodies against several members of the Simbu serogroup, including SHAV [20]. Still, the antibody test can only indicate that the horse has been exposed to a simbuvirus, but cannot confirm whether an infection of the central nervous system is present.

Before SHAV was detected in Europe in 2026, there had been no reports of infections in horses, even though SHAV had been circulating in other regions of the world for decades [21]. When comparing the clinical course of the cases described here with other reports of SHAV in horses in Europe, we find that both horses in our case report exhibited clinically noticeable neurological signs, although these manifested differently in both horses. Similarly, the horses examined in Switzerland exhibited balance disturbances and drowsiness, as described for Horse B [7]. In addition, hypersensitivity to touch and other stimuli was reported, which is consistent with the seizure episodes observed in Horse A [7]. However, apart from the press releases from the Universities of Bern and Zurich regarding horses with neurological abnormalities, as well as the report of a case in Rhineland-Palatinate, Germany, there are currently no known reports of SHAV causing neurological signs in horses [5,7]. Nevertheless, another related virus belonging to the Simbu serogroup, namely SHUV, has been reported to cause neurological signs in cattle and horses [8,9]. The clinical signs described in affected horses from South Africa included fever, ataxia, and episodes of muscle spasms, and are thus partially consistent with the clinical signs of SHAV infection in horses [7,8].

The reported SHUV-induced neurological cases in cattle involved young animals (5 and 16 months old) in which no abnormalities were detected during macroscopic examination of the brain [9], which is in contrast to the hemorrhages observed in the brainstem and thoracic segment of the spinal cord of horse B. However, histological examination revealed perivascular cuffs in the brainstem and cerebrum, with neutrophil involvement in both animals [9]. In addition, cerebral hemorrhages were observed histologically in one cattle [9]. Perivascular cuffing and hemorrhages were also found in these brain regions in our horse cases; however, they were significantly more pronounced in the brainstem and exhibited a different cellular composition of the inflammation (lymphohistiocytic). Furthermore, in one of the cattle, infiltrates were found in the cerebellar meninges, similar to those observed in our horses, though with a different composition of inflammatory cells. In addition, gliosis was found in both cattle; one animal exhibited cerebral neuronal necrosis with neuronophagia as well as aggregates of lymphocytes and neutrophils. The histologic lesions described for SHUV in cattle with neurological signs thus resemble the changes we observed in terms of the localization of the lesions; however, the severity and cellular composition of the inflammation differ. In an experimental study of cattle infected with SHUV showing no clinical neurological signs, the animals developed non-suppurative meningoencephalitis with gliosis, which resembles the changes observed in our horses in regard to lesion and cellular composition [22].

Another factor that must not be overlooked is that both horses exhibited erosions on the head, indicating trauma, likely related to the neurological signs described. Therefore, in the differential diagnosis—particularly for the pronounced hemorrhages in the spinal cord with only mild perivascular lymphohistiocytic infiltration—a traumatic etiology with resorptive inflammation must also be considered. Nevertheless, perivascular infiltrates without hemorrhages were also found in that area, which argues against a purely traumatic etiology.

An important open question is whether SHAV infection may also cause neurological disease in other susceptible species, such as cattle or sheep, or whether milder neurological manifestations occur that currently remain unrecognized, similarly to what was observed in infection experiments with SHUV in cattle. Although the cattle in this study did not show any neurological signs, four of the six infected cattle developed a non-suppurative encephalitis [22]. It will, therefore, be essential to closely monitor such cases, develop and validate improved serological assays for the retrospective detection of SHAV-specific antibodies, and perform experimental infection studies in ruminants to better define the clinical spectrum, tissue tropism, and pathogenesis of SHAV infection.

## 5. Conclusions

We report on two cases in which SHAV could be detected in the CNS tissue of horses, associated with severe neurological signs and histopathological changes. The detection of SHAV in the cerebrospinal fluid is only conclusive following in vivo sampling and in positive cases. The viral load in cerebrospinal fluid is very low, as seen in Horse B, or not detectable at all, as seen in the horses in Switzerland [7], making ante-mortem diagnosis a highly challenging process. Postmortem, CNS material is very suitable for diagnostic purposes. A high viral RNA load and clearly visible pathohistological changes can be found in the brain tissue, particularly in the brainstem.

Overall, the prevalence of SHAV in horses should be further investigated, as the epidemiological role of horses remains unclear. It is imperative to investigate the frequency with which equines become infected and the probability that they will subsequently develop neurological disorders and under which circumstances infections remain asymptomatic. Further studies on the duration of viremia and the epidemiological situation among *Culicoides* biting midges could provide important insights into potential transmission dynamics and serve as a basis for the knowledge of appropriate preventive measures.

## Figures and Tables

**Figure 1:**
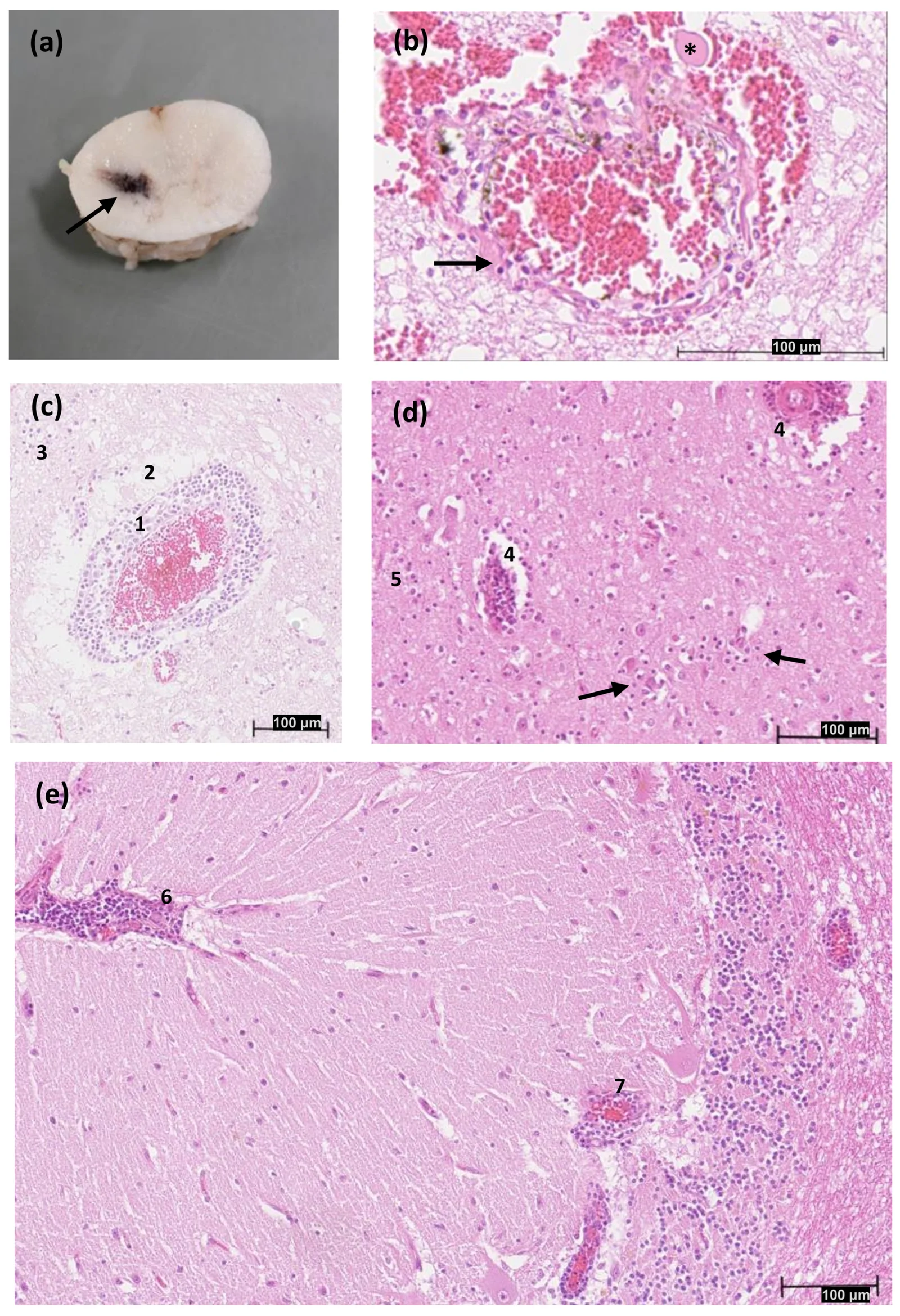
(**a**) Formalin-fixed transverse section through the spinal cord of the thoracic spine, horse B, showing pronounced unilateral hemorrhages (arrow), particularly in the area of the substantia grisea. (**b**) Thoracic spinal cord, H.E., horse B, demonstrating mild lymphohistiocytic perivascular and vascular infiltration (arrow), as well as adjacent hemorrhages and perivascular accumulation of homogeneous eosinophilic proteinaceous material (*). (**c**) Brainstem, H.E., horse A, showing severe lymphohistiocytic perivascular inflammation (1), as well as surrounding edema (2) and lymphohistiocytic infiltration in the adjacent parenchyma (3). (**d**) Brainstem, H.E., horse B, showing lymphohistiocytic perivascular inflammation (4), infiltration of inflammatory cells in the adjacent parenchyma (5), neuronophagia (arrows), and gliosis. (**e**) Cerebellum, H.E., Horse B, showing moderate lymphohistiocytic leptomeningeal infiltration (6) and moderate lymphohistiocytic perivascular infiltration (7) with infiltration of the adjacent neuropil.

**Table 1:** Results and quantification cycle (cq) values of the Sathu-N3-RT-qPCR for the different sample types of Horses A and B. All samples tested negative using the virotype SBV 2.0 RT-PCR Kit (Indical). n.t. = not tested. CSF = cerebrospinal fluid.

	Horse A		Horse B	
Sample	Results	Cq Value	Results	Cq Value
Blood EDTA	n.t.		Negative	
Brain Tissue Pool	positive	17.99	Positive	23.23
CSF	n.t.		Positive	38.13
Brainstem	n.t.		Positive	21.07
Cerebrum	n.t.		Positive	21.62
Cerebellum	n.t.		Positive	24.55
Spinal Cord (Cervical)	n.t.		Positive	29.38
Spinal Cord (Thoracic)	n.t.		Positive	30.32
Spinal Cord (Lumbar)	n.t.		Positive	33.99
Lung	n.t.		Negative	
Heart	n.t.		Negative	
Liver	n.t.		Negative	
Spleen	n.t.		Negative	
Kidney	n.t.		Negative	

## Data Availability

The original contributions presented in this study are included in the article/Supplementary Material.

## Supplementary Materials

The following supporting information can be downloaded at https://www.mdpi.com/article/10.3390/v18101125/s1. Table S1: Positive results in cattle in municipalities adjacent to Horse A; Table S2: Positive results in cattle in municipalities adjacent to Horse B.
